# An Overview of Advanced Antimicrobial Food Packaging: Emphasizing Antimicrobial Agents and Polymer-Based Films

**DOI:** 10.3390/polym16142007

**Published:** 2024-07-13

**Authors:** Punita Upadhyay, Muhammad Zubair, M. S. Roopesh, Aman Ullah

**Affiliations:** Department of Agricultural, Food, and Nutritional Science, University of Alberta, Edmonton, AB T6G 2P5, Canada; pupadhya@ualberta.ca (P.U.); mzubair1@ualberta.ca (M.Z.); roopeshms@ualberta.ca (M.S.R.)

**Keywords:** antimicrobial agents, active food packaging, shelf-life extension, food preservation

## Abstract

The food industry is increasingly focused on maintaining the quality and safety of food products as consumers are becoming more health conscious and seeking fresh, minimally processed foods. However, deterioration and spoilage caused by foodborne pathogens continue to pose significant challenges, leading to decreased shelf life and quality. To overcome this issue, the food industry and researchers are exploring new approaches to prevent microbial growth in food, while preserving its nutritional value and safety. Active packaging, including antimicrobial packaging, has gained considerable attention among current food packaging methods owing to the wide range of materials used, application methods, and their ability to protect various food products. Both direct and indirect methods can be used to improve food safety and quality by incorporating antimicrobial compounds into the food packaging materials. This comprehensive review focuses on natural and synthetic antimicrobial substances and polymer-based films, and their mechanisms and applications in packaging systems. The properties of these materials are compared, and the persistent challenges in the field of active packaging are emphasized. Specifically, there is a need to achieve the controlled release of antimicrobial agents and develop active packaging materials that possess the necessary mechanical and barrier properties, as well as other characteristics essential for ensuring food protection and safety, particularly bio-based packaging materials.

## 1. Introduction

Annually, approximately 1.3 billion metric tons of food are wasted around the world [1], which represents a significant economic loss for the food industry. The primary cause of these food losses is microbial spoilage of food products. Recently, outbreaks of foodborne illnesses have posed significant threats to public health and put pressure on the food industry and policymakers. Contamination by microorganisms is the main cause of food loss [2,3], and it also changes the nutritional value and sensory features of foods. These changes are a result of oxidation, which affects the odor, taste, appearance, texture, and color of food [4].

There is a pressing need to develop innovative technologies that provide consumers with high-quality food products. Food packaging technologies are critical in this regard, as they have traditionally been designed to ensure the safe and secure transportation, delivery, and consumption of food. The primary functions of food packaging include protecting, controlling, containing, easing, and communicating the packaged food. Specifically, packaging serves to safeguard against adverse environments, such as sunlight, gases (O_2_ and CO_2_), water vapor, mechanical strength, and microorganisms [5,6]. The prime function of food packaging is to safeguard food from external elements by preventing cross-contamination. Food packaging plays a partial or complete role in preventing physical harm, slowing down or stopping decay, and extending the shelf life of food products, while maintaining their quality. However, microbial growth, which reduces the quality and shelf life of food, is the main cause of food spoilage. Spoilage changes the natural microflora in food, leading to pathogenic hazards for consumers [7,8]. Various microorganisms, including bacteria, yeast, and molds, can cause microbial spoilage of packaged food products. Nevertheless, their ability to degrade food depends on several factors, such as the nature of the nutrients, pH of the food and environment, water activity within the packaged system, and the presence of gases. Thus, the contamination of food products depends on various pathogenic microorganisms and environmental conditions, resulting in the complete spoilage of food or its prevention.

Numerous food processing techniques can be employed to either inactivate or prevent the growth of pathogens in food products and to avoid cross-contamination. Conventional methods like thermal processes (e.g., sterilization, pasteurization, and blanching) and non-thermal technologies (e.g., irradiation, pulsed electric fields, and high-pressure processing) have been traditionally used to eliminate pathogens from food items [9,10]. However, these methods cannot completely prevent contamination and/or the growth of microorganisms. This issue can be resolved by implementing food packaging, particularly active food packaging techniques. In this regard, incorporating natural antimicrobial compounds into packaging materials produces improved packaging technology. Active food packaging is an advanced technique in which important data about the freshness or spoilage of food inside a package are communicated to consumers [11]. This packaging technique involves the incorporation of different active agents into food packaging materials to improve their functional properties and prevent microbial spoilage of food. The active agent within the packaging material serves two functions: firstly, interacting with the food product, and secondly, protecting the space between the packaging material and the food, thereby safeguarding it from microbial attack [12]. An effective strategy for extending the shelf life of food products involves the incorporation of active ingredients into both natural and synthetic polymers, along with the advancement of coatings and films. Employing renewable and biodegradable polymers instead of synthetic polymeric materials offers several benefits and helps to protect the environment by reducing human-generated plastic pollution [13,14]. Using antimicrobial packaging solutions, sustainable active packaging can meet industry standards for safety, quality, and extended shelf life. Although the incorporation of antimicrobial substances into packaging materials has been extensively studied, the challenge lies in controlling the release of antimicrobial substances and enhancing their effectiveness in food products.

This review provides a comprehensive overview of current research and emerging technologies in the field of antimicrobial food packaging, with a specific focus on the use of antimicrobial agents and polymer-based films. By highlighting innovative aspects and addressing recent advancements, this review seeks to differentiate itself from the existing literature and offer new insights into effective strategies for extending the shelf life of food products. 

## 2. Antimicrobial Food Packaging 

Antimicrobial packaging is an innovative method with a promising future owing to high consumer demand for safe and high-quality food products. The primary purpose of using antimicrobial agents in packaging materials is to enhance food safety and extend the shelf life of food products. Antimicrobial agents achieve these desired functions by slowing down or hindering the growth of pathogens in packed food or packaging materials [15]. Fruit, vegetables, dairy, meat, and bakery products are easily spoiled by microorganisms and can be protected by antimicrobial packaging [16].

The addition of antimicrobial compounds to the packaging material or its surface coating suppresses the growth of targeted microorganisms that contaminate food products [17]. The main purposes of conventional food packaging are the extension of shelf life, maintenance of quality, and safety and security assurance of food products. On the other hand, the primary functions of antimicrobial packaging are in reverse order as compared to traditional packaging [5].

**Figure 1:**
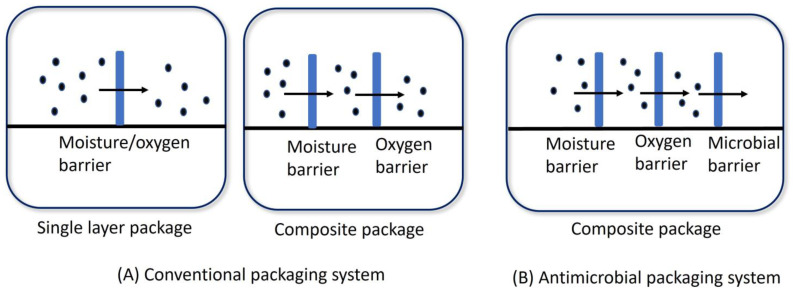
Barrier technology in antimicrobial packaging systems compared to conventional packaging (modified from [5]).

All antimicrobial agents have different modes of action on pathogenic microorganisms because of their specific mechanisms of action and physiologies. The selection of an antimicrobial agent to target a specific microorganism depends on the categorization of the microorganism, such as its oxygen requirements, cell wall composition, growth stage, and optimal growth temperature. In addition to microbial properties, the specific antimicrobial action of an antimicrobial substance is critical for distinguishing its efficiency and the optimum activity for microbial decontamination. Antimicrobial agents mainly act in two ways: by inhibiting the essential metabolic/reproductive genetic pathways of microorganisms, or by altering cell membrane/wall structures [18]. The fundamental mode of action of antimicrobial packaging is based on hurdle technology, as shown in Figure 1. 

Antimicrobial packaging is being used to prevent the growth of microorganisms while maintaining the traditional role of packaging as a barrier to water vapor and gas. This is achieved by incorporating antimicrobial agents into packaging systems or polymeric materials using one of three methods: release, absorption, or immobilization [19]. During the release mode, antimicrobial agents migrate into the food or headspace inside the packaged food and prevent microbial growth. Most commonly, solutes or gases can be the antimicrobial agents in this case. In the absorption mode, antimicrobial substances remove water vapor and gases (oxygen and carbon dioxide) and control the changes in pH within the system, which can help prevent microbial growth. Carbon dioxide and oxygen scavengers, ethanol emitters, and moisture absorbers are used as absorbents to control the growth of bacteria and molds in food packages. The immobilization-based antimicrobial packaging systems do not release any antimicrobial agents within the system. Instead, the growth of microorganisms is inhibited at the contact surface. The application of immobilized lysozyme and glucose oxidase enzymes to the polymer packages of cheese, beef, and culture media has been reported. It is essential to mention that the immobilization system is more suitable for liquid foods in comparison with solid foods. This can be ascribed to the lack of direct contact between antimicrobial packages and solid food products. 

## 3. Antimicrobial Packaging Construction

### Antimicrobial Mechanism

Every antimicrobial material has a unique mode of inhibition and mechanism of action against specific microorganisms. Therefore, the selection of antimicrobial agents depends on their specific efficacy against target microorganisms. There is no single antimicrobial agent which can work against all pathogenic microorganisms. The chemical structure and functionality of the antimicrobial material and physiological differences in the microorganism structure play key roles. Microorganisms can be classified based on their cell wall composition (i.e., gram-positive or gram-negative), the nature of the cells (spores/vegetative), oxygen requirements (aerobic or anaerobic microorganisms), and optimal temperature for growth (psychrophiles, mesophiles, thermophiles, or hyperthermophiles). These criteria are an excellent way to select an antimicrobial agent for a specific microorganism. The importance of antimicrobial agent efficiency and concentration limit is as vital as considering the physical attributes or growth circumstances when targeting a specific microorganism. Some antimicrobial agents hinder essential metabolic or reproductive genetic pathways, whereas others alter the structure of microbial cell walls. For instance, chitosan modifies the cell walls of microorganisms. Chitosan is a natural polymer composed of ß-1,4-linked glucosamine and N-acetyl glucosamine, which is commonly synthesized through the deacetylation of chitin [20,21].

**Figure 2:**
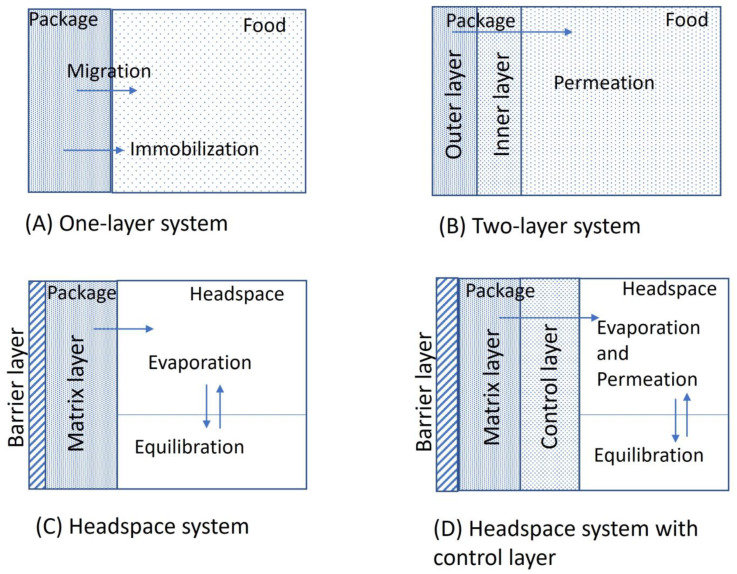
Antimicrobial packaging systems with their releasing profiles (modified from [5]).

Chitosan exhibits improved antimicrobial properties when it possesses a positive charge, which allows greater solubility [21,22], ascribed to the protonated amino group. Thus, it can interact with microbial cell membranes, which are negatively charged, leading to the leakage of their intracellular components [23]. It is widely recognized that chitosan is released from the fungal cell wall due to plant host hydrolytic enzymes [24]. Antimicrobial agents can be introduced into food packaging systems by blending, immobilization, or coating. However, this depends on the nature of the packaging materials (petroleum or bio-based), antimicrobial agents, and packaged food. In the blending of antimicrobial agents, it can migrate from packaging materials to the food product. However, in the case of immobilization, the antimicrobial agents cannot migrate. Antimicrobial systems and their releasing mechanisms are explained in Figure 2, where in a one-layer system, antimicrobial agents are combined or chemically attached to the packaging material using immobilization, while in a two-layer system, an antimicrobial agent is coated on the outer layer of the packaging material, or the antimicrobial matrix layer (outer layer) is covered with the inner layer (control layer) to control the release of the antimicrobial agent. The third type is the headspace system, in which the antimicrobial agent is volatile, first introduced into the matrix, and then released into the packaged food headspace. The fourth is also a headspace system; however, in this case, a control layer is established, which controls the permeation of volatile antimicrobial agents while maintaining a particular concentration in the headspace region. The first two systems use the phenomenon of diffusion for the release of the antimicrobial agent, while the last two methods use the evaporation process to release volatile antimicrobial agents into the headspace.

## 4. Composition of Antimicrobial Packaging Materials

The addition/incorporation of antimicrobial agents in the headspace, which is a non-food part, is a package or package in a gaseous form. Directly incorporated antimicrobial agents in packaging materials are in different forms, such as films, overcoated films, sheets, trays, and containers, or inside packages such as sachets, pads, or inserts.

**Figure 3:**
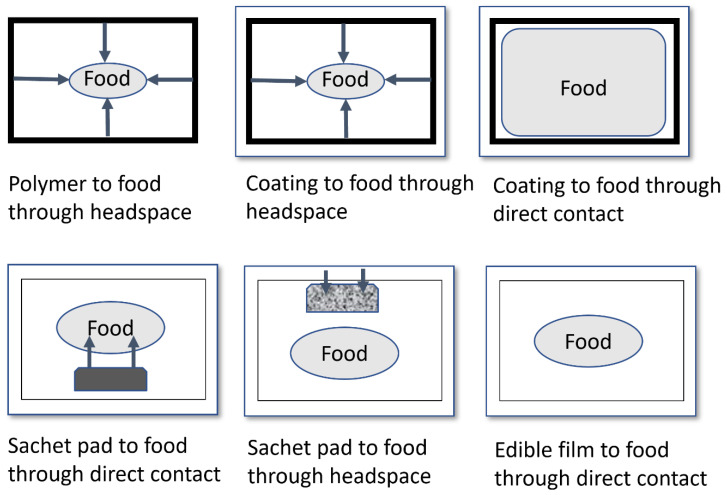
Potential methods of antimicrobial release in food packaging applications [18,25,26].

Edible antimicrobial agents containing edible coatings on food can prevent microbial degradation [5]. Figure 3 shows the potential methods for antimicrobial release in food packaging applications, such as polymer to food through headspace, coating of food through headspace, coating of food through direct contact, sachet pad to food through direct contact, sachet pad to food through headspace, and edible film to food through direct contact. 

Antimicrobial packaging can be constructed in different forms, including packages with sachets or pads with volatile antimicrobial agents, polymer matrices incorporated with volatile and nonvolatile antimicrobial agents, and polymer surfaces coated with antimicrobials. Immobilization of antimicrobial agents to polymers occurs by ionic or covalent bonds and the use of inherently antimicrobial polymers.

### 4.1. Antimicrobial Packaging Material

Antimicrobial packaging is classified into two main categories: non-biodegradable and biodegradable packaging.

#### 4.1.1. Non-Biodegradable Packaging

Most of the materials used in food packaging are composed of non-degradable plastics because of their advantageous properties, such as low cost, light weight, and flexibility. They are obtained from non-renewable petroleum-based natural resources. The most commonly used non-biodegradable polymers in food packaging are polyvinyl chloride (PVC), high-density polyethylene (HDPE), low-density polyethylene (LDPE), polypropylene (PP), polyethylene-co-vinyl acetate (EVA), and polyethylene terephthalate (PET), etc. Polyvinyl chloride (PVC) is the most commonly used synthetic polymer/plastic material in the food packaging industry owing to its ease of use in obtaining desirable materials with antimicrobial properties. These films have many processing benefits, such as low cost, wide availability, high average strength, flexibility, ease of heat sealing, chemical inertness, high permeability, and exceptional self-sticking properties [27].

High-density polyethylene (HDPE) is a thermoplastic ethylene polymer with some branches. HDPE is rigid, strong, and has better water vapor barrier properties, making it less transparent than LDPE. These films are more durable and can keep food fresh for a longer time, which means extending the shelf life of food when used as a food packaging material.

Low-density polyethylene (LDPE) is a popular plastic polymer used in food packaging. LDPE is very cheap compared to other plastic materials/films. As a food packaging material, LDPE is transparent and can strongly resist water vapor, but cannot control oxygen, carbon dioxide, and a few other gaseous compounds. LDPE films exhibit good heat-sealing properties and can be used for sealing [28].

Polypropylene (PP) is synthesized via propylene chain-growth polymerization. It is a thermoplastic resin with a low density (0.89–0.91 g/cm^3^) [27] compared to other plastic polymers. PP has a high melting point and strong water vapor resistance, making it suitable for high-temperature packaging operations.

Polyethylene-co-vinyl acetate (EVA) films are widely used as refrigerated items in food packaging. This film is prepared by the copolymerization of polyethylene (PE) and polyvinyl alcohol (PVA) with shallow gas or vapor permeability [29]. EVA films are commonly used in food packaging because of their nontoxicity, flexibility, high adhesivity, good stretching, and easy heat-sealing properties [30,31].

Polyethylene terephthalate (PET) is a transparent thermoplastic prepared using ethylene glycol and dimethyl terephthalate. PET films are an excellent choice as food packaging materials over other standard plastic films because of their high tensile strength, good mechanical properties, good stability, and high melting point. PET is mainly used as bottle-making material because of its safety and easy processing [32].

#### 4.1.2. Biodegradable Packaging

Although the most commonly used materials in food packaging are non-degradable plastics, their use is decreasing because of their non-biodegradability. It causes immense environmental pollution and poses a significant threat to humans and wildlife. Biodegradable food packaging materials are the most promising and excellent alternatives to conventional packaging because they are sustainable, renewable, green, and environmentally friendly. In biodegradable packaging, the degradation process occurs naturally by the activity of microorganisms, such as bacteria, fungi, and algae. Nowadays, biodegradable antimicrobial packaging materials and their uses are concomitant with the evolution of biodegradable polymers such as polylactic acid (PLA), cellulose, proteins, lipids, chitosan, and starch.

Polylactic acid (PLA) is recognized as one of the most eco-friendly biopolymers because of its renewability, biodegradability, biocompatibility, and excellent physical and chemical properties [33]. The fermentation process derives PLA from renewable resources. It is prepared by the condensation polymerization of lactic acid or the ring-opening polymerization of lactide in the presence of a catalyst [34]. Polylactic acid is approved as a safe biopolymer for food packaging material by the US Food and Drug Administration (USFDA). PLA-based antimicrobial materials are promising systems for controlling microbial growth. Polylactic acid-based films provide better results against microbial growth than other antimicrobial films because of their competitive price and eco-friendliness. However, one of the significant disadvantages is the poor barrier properties, such as high gas- or vapor-permeability, limiting its use in food packaging [35].

Cellulose, mainly obtained from plant wood, is the most abundant natural biopolymer. It is a biodegradable, biocompatible, and environmentally friendly linear homopolysaccharide [36]. Composite films made from cellulose have attracted considerable attention for use in food packaging because of their biodegradability and recyclability. However, their commercial applications are limited due to their high cost and sensitivity to water.

Starch is one of the most explored biopolymers because it is abundantly available, cheap, non-toxic, biodegradable, and biocompatible as a food packaging material. It has the advantage of acting as a moderate barrier to oil. Still, it has remarkable moisture sensitivity and poor mechanical properties due to hydrophilic functional groups in its molecular structure, as compared to conventional packaging, limiting its applications in food packaging.

Chitosan ß-1,4-linked glucosamine and N-acetyl glucosamine are synthesized by deacetylation from chitin. They are natural biopolymers with linear structures and are non-toxic, biodegradable, and biocompatible, with broad spectra of antimicrobial activities [37]. Chitosan has natural antimicrobial properties and can be used as an antimicrobial agent and a polymer matrix/substrate simultaneously. Because of their excellent antimicrobial properties, chitosan films can be used to preserve fresh food for a longer period and extend the shelf life of food [38]. Chitosan can be obtained by extrusion and compression molding techniques, making it easy to demonstrate its antimicrobial properties in packaging films. Chitosan–polyvinyl alcohol blends possess the advantages of antimicrobial activity against food pathogen bacteria due to biological compatibility [39].

Besides biodegradable biopolymer materials, proteins, lipids, polysaccharides, and other edible biopolymers, food additives are also good choices for producing edible antimicrobial films.

#### 4.1.3. Antimicrobial Agents

There are different types of microorganisms (bacteria, yeasts, and molds) that degrade and spoil foods and lower their quality and shelf life. Many of these microorganisms may be prevented by using various antimicrobial agents directly added to conventional food packaging materials or systems to achieve new antimicrobial packaging materials or designs. These antimicrobial agents may be used alone or be incorporated into a polymeric matrix to obtain more effective antimicrobial properties. Antimicrobial agents improve the quality and maintain the safety standards of food packaging by reducing surface contamination and inhibiting the growth of microorganisms in processed foods. However, antimicrobial agents should not be used as alternatives to good sanitization practices and hygiene [40]. Different types of antimicrobial agents are added or incorporated into food packaging systems and are classified as chemical antimicrobials, natural antimicrobials, and antioxidant probiotic antimicrobial polymers. Table 1 shows some potential biopolymer antimicrobial agents for food packaging systems.

Antimicrobial agents can be classified into three main groups: chemical antimicrobial agents, natural antimicrobial agents, and probiotics [15,18].

Chemical antimicrobial agents can be used in different ways: blended with food additives, incorporated into packaging materials, or inserted into the headspace of food packaging. Organic acids are most commonly used because they are cost-effective and their efficacy is well understood. Organic acids and their salts, sulfites, nitrites, antibiotics, and alcohols are widely used as synthetic antimicrobial agents, showing characteristic sensitivities towards microorganisms. Organic and inorganic materials or salts are commonly used in antimicrobial food packaging [43]. Organic acid salts, such as sodium benzoate and potassium sorbate, are generally considered safe and they are also widely used worldwide as preservatives. Potassium sorbate is the most powerful preservative against fungi and is potentially used in bakery products [44]. Numerous inorganic compounds and their nanoparticles, such as silver, copper, zinc, and titanium oxide, exhibit antimicrobial activity when incorporated into packaging systems. Silver zeolite-filled polypropylene composites were utilized to impart antimicrobial properties to the polymeric films. [45]. Titanium oxide exhibited antimicrobial activity similar to that of silver after obtaining a photocatalytic effect [46]. Zinc nanoparticles (NPs) are antimicrobial/antibacterial agents used in food packaging against foodborne diseases. They offer strong antimicrobial activity against foodborne pathogens by affecting cell wall permeability, cellular respiration, and respiration [47].

Natural antimicrobial agents contain plant spices/herb extracts, enzymes, and bacteriocins which are naturally occurring antimicrobial agents. Nowadays, people prefer chemical and preservative-free foods, which makes naturally occurring antimicrobials in high demand to purify food and prolong shelf life. Plant herb/spice extracts contain many naturally occurring compounds with a broad range of antimicrobial spectra against different microorganisms [48]. Naturally occurring antimicrobial agents also have antioxidant properties that contribute to some medicines. However, their chemical stability, kinetics, and mechanisms of action are unknown. The antimicrobial activity of enzymes depends on the environmental conditions. Lysozyme is too sensitive to environmental temperature and pH, which makes it less or almost ineffective against gram-negative bacteria. Bacteriocins are small molecules formed by bacteria that prevent the growth of similar or closely related bacterial strains. Various bacteriocins, such as lacticin, nisin, and EDTA, have been directly added to edible films, coatings, and plastic films [49]. Other bacteriocins, such as pediocin and propionicin, are incorporated into food or food packaging systems to prevent the growth of microorganisms. Bacteriocins produced by live bacteria occur as a result of the fermentation of food products added to food packages as probiotics to obtain effective antimicrobial properties. Immobilized bacteriocins nisin and lacticin are incorporated into polyethylene/polyamide pouches to protect against *Lactococcus lactis*, *Listeria innocua*, and *Staphylococcus aureus* in cheese and ham stored at refrigerator temperatures, thereby increasing shelf life [50].

Probiotics are obtained from many microorganisms like lactic acid bacteria, and can prevent the growth of other harmful bacteria such as the Gram-negative bacteria *Escherichia coli*. Currently, limited information is available on probiotics for use in antimicrobial food packaging design systems. However, probiotics may be of interest in the near future because of their benefits and effectiveness against bacteria. Different antimicrobial agents and their applications in food packaging systems are shown in Table 2.

#### 4.1.4. Methods for Incorporating Antimicrobial Agents into Matrices

There is a lot of research conducted on antimicrobial food packaging; however, so far, the progress is not sufficient to prevent the growth of microorganisms and increase and maintain products’ shelf life. Most of the antimicrobial agents used are highly sensitive to the conditions of the film-making process under high temperatures and pressures. The deformation or evaporation of volatile antimicrobial agents occurs under processing conditions such as high temperatures and pressures [85]. However, among plastic manufacturing technologies, blown film extrusion and compression molding are frequently used as heat-stable antimicrobial methods that require mild processing conditions. For rapid processing, it is critical to apply other methods under elevated conditions to reduce the loss of antimicrobial agents. Using a conventional method that involves very high temperatures, scientists have found a significant reduction in the use of antimicrobial agents. Ramos M. et al. reported in their article that 25–44% weight of thymol and carvacrol remained in polypropylene (PP) film when in a hot-press process, with the temperature applied at 190 °C for 18 min. They also reported that antimicrobial agents were only effective against *S*. *aureus* when using a high initial concentration of 8% *w*/*w*. They also mentioned that after the hot-press method, the antimicrobial agent retained only 3.5% *w*/*w* [86].

An alternative method incorporating volatile antimicrobial agents has been studied to prevent the loss of antimicrobial agents during film processing. A few researchers have developed a masterbatch of ethylene-vinyl acetate (EVA)/antimicrobial compounds at comparatively low temperatures before undergoing the film production process [87]. In the blown film-making process, EVA is first mixed with antimicrobial agents to obtain homogeneous mixtures and then combined with LDPE powder at room temperature before undergoing the procedure. Some researchers have demonstrated that ethylene-vinyl acetate has successfully absorbed a large amount of volatile antimicrobial agents, which is attributed to the polarity of the hydroxyl group in EVA towards antimicrobial agents, which exhibit varying degrees of polarity. They also researched the released kinetic energy of antimicrobial agents from EVA/LDPE film. They found that the optimum concentration weight of EVA in LDPE to obtain the minimum release rate of antimicrobial agents was 10%.

Heat sensitive antimicrobial agents and volatile compounds are preferably obtained by non-heating methods like electrospinning, surface coating, and solvent compounding. Surface coating is the most popular method for applying antimicrobial agents to polymer surfaces because of its simple processing. To obtain efficient affinity of antimicrobials for the film matrix, films typically undergo the process of surface modification by using corona or UV radiation treatment before the application of antimicrobial agents; usually, this entire process is performed at room temperature. Studies on antimicrobial-coated films have used chitosan/essential oil-coated polypropylene films [88], chitosan/bacteriocin-coated plastic films [89], bacteriocin-coated LDPE films [90], oregano essential oils, and citral-coated PP/EVOH films [90].

Interestingly, antimicrobial films prepared at high temperatures demonstrate better prevention than the surface coating method against microorganisms [91]. Researchers prepared antimicrobial films using the extrusion method, compared them with ion-coated antimicrobial film with the incorporation of the same amount of antimicrobial agent, and reported that this method is more effective against *E. coli*, *Salmonella Typhimurium*, and *Listeria monocytogenes*. The results indicated that the extrusion method permitted more effective incorporation of the active compounds into the polymer matrix. However, there has been insufficient research comparing both approaches; therefore, an effective comparison still needs to be made in the future for further exploration.

The film casting method is another method used to stop the loss of antimicrobial agents. It is usually conducted at a much lower temperature and does not depend on mechanical shear forces. Generally, this method is limited to natural polymers with a low melting temperature. The film uses natural biopolymers, such as chitosan, starch, and alginate, which are usually biodegradable and edible.

## 5. Types of Antimicrobial Packaging

Antimicrobial packaging consists of many forms, such as (i) sachets and pads containing volatile and non-volatile antimicrobial agents added into packages; (ii) volatile and non-volatile antimicrobial agents directly incorporated into polymers; (iii) antimicrobial coating onto polymer surfaces; (iv) immobilization of antimicrobial agents onto polymers by ionic or covalent linkages; and (v) use of inherently antimicrobial polymers [15].

### 5.1. Sachets or Pads Containing Antimicrobial Agents Added into Packages

Sachets are used in antimicrobial packaging enclosed or loose inside the package. Oxygen absorbers, moisture absorbers, and ethanol vapor generators are three types of sachets that are widely used as antimicrobials [15]. Oxygen and moisture absorbents inhibit microbial growth by lowering water activity, preventing oxidation, and restricting the growth of yeast and mold in meat, pasta, and bakery packaging [92]. In bakery and dried products, ethanol vapor-containing sachets are used to prevent the growth of mold [93].

### 5.2. Antimicrobial Agents Directly Incorporated into Polymers

Antimicrobial agents incorporated into polymer matrices have commercial applications in pharmaceuticals, surgical implants, pesticide delivery, textiles, and other household goods. Table 3 shows a few food-related commercialized applications that incorporated antimicrobials to inhibit the surface growth in food packages. The enzymatic activity of microbial cells decreases when zeolites are substituted with 1–3% silver incorporated into polypropylene, polyethylene, nylon, and butadiene-styrene. Chelating agents such as EDTA used in the preparation of edible films incorporated with nisin or lysozymes can prevent the growth of *E. coli* [94].

### 5.3. Coating or Adsorbing Antimicrobials to Polymer Surfaces

Wax-coating with fungicides keeps fruits and vegetables fresh for a longer period, increasing their shelf life, so films incorporated with quaternary ammonium salt coats were used to wrap potatoes [97]. Another early development of coated antimicrobials was the wrapping of sausages and cheese with wax paper and cellulose casing coated with sorbic acid [98]. Antimicrobial agents are not suitable for the high temperatures used in polymer processes and are coated onto the material after being added to cast films. Polyethylene films coated with nisin and methylcellulose [40] and zein and nisin were used for poultry [15].

### 5.4. Antimicrobials Ionically or Covalently Immobilized to the Surface of the Polymer

The polymers and antimicrobials used for immobilization techniques require functional groups for antimicrobial packaging. Functional groups of antimicrobial agents include polyamines, peptides, enzymes, and organic acids. In contrast, polymers with functional groups include ethylene methyl acrylate, ethylene vinyl acetate, ethylene acrylic acid, ethylene methacrylic acid, nylon, polystyrene, and ionomer [15]. In addition to functional antimicrobials and polymer matrix supports, immobilization may also necessitate the use of molecules called ‘spacers’ that link bioactive antimicrobial agents to the polymer surface. These spacers provide motion freedom, allowing the bioactive agents to easily reach microorganisms on the surface of the food. Some examples of spacers used in antimicrobial food packaging owing to their low toxicity are polyethylene glycol (PEG), polyethyleneimine, ethylenediamine, and dextrans. Some examples of ionic and covalent immobilization of antimicrobials onto polymers and other materials are shown in Table 4.

### 5.5. Inherent Antimicrobial Polymers

A few polymers are inherently antimicrobial and have been used in coatings and films. Inherent polymers, such as chitosan and poly-L-lysine, are cationic polymers that promote cell adhesion [100]; therefore, the interaction between charged amines and negative charges on the cell membrane causes leakage of intracellular constituents. Chitosan has been used as a coating material to protect fruits and vegetables, keep them fresh, and to prevent fungal degradation. Chitosan-based antimicrobial films have also been used to carry organic acids and spices [101]. Chitosan coatings are mostly applied to vegetables and fruits to protect them from fungi and to act as a barrier between nutrients and microorganisms [102]. Chitosan films and coatings are also used in various chemical and natural antimicrobial agents such as organic acids and spices [101,102]. Inherent polymers such as chitosan and poly-L-lysine inhibit microbial growth by disrupting the leakage of intracellular constituents of microbial cells [15].

## 6. Effectiveness of Antimicrobial Packaging

Numerous studies have shown that antimicrobial packaging can effectively prevent specific microorganisms when specific antimicrobial agents are incorporated into food packaging materials. There are two main reasons why antimicrobial packaging is more effective than the direct addition of preservative agents into the food; the first is the incorporation of antimicrobial agents in the polymer film, which allows the gradual release of antimicrobial function over a more extended period, and the second is that antimicrobial agents added directly into the food as a preservative agent may undergo inactivation, such as hydrolysis, neutralization, dilution, etc., by food matrixes and components [15,90]. In addition, preservative agents directly added to the food may change the taste and texture of the food and drastically reduce the quality of the food. Therefore, antimicrobial packaging plays an important role in preventing the growth of specific types of bacteria on food, simultaneously improving food safety and extending the shelf life of food without compromising the quality of the food. The functionality of antimicrobial agents in foods is an essential factor in the growth of microorganisms. Food is considered spoiled when the total value of the microbial count of 10^7^ CFU/g exceeds 10^8^ CFU/g; above this level, it starts giving off an unpleasant odor. Most researchers who explored antimicrobial packaging referred to a bacterial count of 10^7^ CFU/mL or gm or cm^2^ as a standard for shelf life indication.

Several studies have been conducted regarding the effectiveness of antimicrobial packaging when essential oils and plant extracts, enzymes, chitosan, bacteriocin, and inorganic materials are used in the packaging. Nowadays, inorganic nanoparticles have gained much attention for antimicrobial food packaging because of their nanoscale size, high surface area, and high volume ratio, which enhance their surface reactivity [103].

### 6.1. Antimicrobial Packaging of Plant Extracts and Essential oils

Many studies have been performed on plant extract components in food packaging, examining their effectiveness. Plant extracts and essential oils are well-known for their antimicrobial activities, and numerous studies have reported the antimicrobial activities of plant essential oils against foodborne pathogenic microorganisms. Essential oils are rich in phenolic compounds and volatile terpenoids; therefore, they have a very high potential to prevent a wide spectrum of microorganisms. Talebi et al. prepared an antimicrobial polylactic acid (PLA) film incorporated with different concentrations of Mentha piperita essential oil (MPO), Bunium percicum essential oil (BPO), and nanocellulose (NC). They used the prepared films to seal ground beef for 12 days and stored it at 4 °C. They reported that Mentha piperita essential oil (MPO) and Bunium percicum essential oil (BPO) had organoleptic and antimicrobial activities against *Enterobacteriaceae*, *Staphylococcus aureus*, and *Pseudomonas*. Among all the different concentrations, they found the films with the most effective antibacterial and organoleptic properties were those with concentrations of PLA, MPO, and NC of 1% *w*/*v*, 0.5% *v*/*v*, and 1% *v*/*v,* respectively [104].

Issa A. et al. prepared active biodegradable nanocomposite films from sweet potato starch and montmorillonite (MMT) nano clay incorporated with thyme essential oil (TEO) for food packaging. The data indicated that after essential oils are mixed, within five days, *E. coli* and *Salmonella Typhimurium* volume levels were reduced to a significant level (*p* < 0.05), while the control (without essential oils) group contained approximately 4.5 (CFU)/g [105].

### 6.2. Bacteriocins

Bacteriocins are peptide antimicrobials that are biologically derived from bacterial microorganisms. Bacteriocins are produced from various sources with different structures, which are favorable for the development of bacteriocins and act as new antimicrobial agents. Bacteriocins are used to control a specific type of bacterial growth in food, maintain food quality safety, and improve the shelf life of food products. Most of the time, they are produced by lactic acid-producing bacteria. Nisin and pediocin are the most commonly used bacteriocins. The most popular antibacterial peptide used in active food packaging is nisin, which is synthesized by lactic acid bacteria (LAB) and *Lactococcus lactis*. Nisin is a U.S. Food and Drug Administration-approved bacteriocin. Nisin showed wide-spectrum effectiveness against Gram-positive microorganisms such as *Listeria monocytogenes* [106,107]. Nisin has both hydrophobic groups and positive charges. The positively charged antimicrobial peptide interacts with negatively charged bacteria and is attached to the bacterial cell membrane [108]. In addition, the hydrophobic amino acid group of the antibacterial peptide can be embedded into the hydrophobic bacterial cell membrane to change its permeability. Nisin can be easily mixed with polymers to demonstrate its antimicrobial properties by incorporating them into polymers or coating them on polymer films to produce films in food packaging. Several studies have reported the effectiveness of nisin-containing active packaging in preventing the growth of various broad-spectrum foodborne bacteria.

Sugandha Bhatia et al. studied the antibacterial effectiveness of starch-based packaging film incorporated with nisin, EDTA, and lysozyme. They reported that their hypothesis that nisin and EDTA have a partial collaborative effect on antibacterial activity was supported [109]. Divsalar et al. prepared a composite film of chitosan/cellulose incorporated with nisin. They tested the effectiveness of the composite in storing ultrafiltered (UF) cheese at 4 °C temperature for 14 days. The control film, which contained only chitosan-cellulose, did not show antimicrobial effectiveness against *L. monocytogenes*, whereas the nisin-incorporated composite film showed a substantial increase in prevention against *L. monocytogenes* [110].

### 6.3. Enzyme-Lysozyme

Lysozyme is a naturally occurring antimicrobial enzyme that contains a hydrophilic monopeptide chain [111] that is remarkably effective against Gram-positive bacteria and can prevent bacterial infections. Lysozymes function as antimicrobials by hydrolyzing the beta-1–4 glycosidic bonds between *N*-acetylmuramic acid and *N*-acetylglucosamine in peptidoglycans [112]. Lysozyme hydrolyzes glycosidic bonds in peptidoglycan, which is the main cell wall component of bacteria, especially Gram-positive bacteria, and destroys the cell wall membrane, causing intracellular materials to leak out, resulting in the death of bacteria [113].

Numerous studies have been conducted on the development of food packaging materials based on antimicrobial enzyme immobilization by physical blending or chemical bonding. Corradini et al. prepared an antimicrobial packaging film using lysozyme via the sol-gel route using poly (ethylene terephthalate) (PET) as a polymer matrix with controlled release properties. The antimicrobial activity of the films was tested against *Micrococcus lysodeikticus*. The results showed that lysozyme migration possibly occurred between the film and water and remained active after incorporation into the PET films [114]. Muriel et al. introduced a novel antimicrobial film on which lysozyme was covalently bonded to two distinct ethylene vinyl alcohol copolymers, denoted EVOH 29 and EVOH 44. The antibacterial effectiveness of the produced films was evaluated against *L. monocytogenes*, showing an efficacy similar to that of the unbound enzyme. Specifically, the log reduction values for EVOH 29-lysozyme, EVOH 44-lysozyme, and free lysozyme are 1.08, 0.95, and 1.34, respectively [111].

### 6.4. Chitosan

Chitosan is a natural biopolymer that has been widely studied because of its biodegradability and biocompatibility. Chitosan has inherent antimicrobial characteristics and is widely used, especially in food packaging; it is also used in the fields of biomedicine and environmental conservation [115]. The excellent film-forming properties and antibacterial activity of chitosan make it the best material for antibacterial food packaging. The effectiveness of antimicrobial properties also depends on the molecular weight of chitosan. Generally, low- and medium-molecular weight chitosan have shown high antimicrobial activity against most bacterial cultures, with mean minimum inhibitory concentration (MIC) values of 0.01 and 0.015% *w*/*v*, respectively [116].

The antimicrobial mechanism of chitosan has been divided into three types [117]: (i) Absorption of chitosan onto the bacterial cell membrane, in which the positive charge of chitosan electrostatically attracts the negatively charged bacterial cell walls, and adsorption of chitosan causes intracellular components to break down to cell death. (ii) Accumulation of chitosan can hide or isolate bacteria to prevent nutritional loss in food. (iii) The antimicrobial activity of chitosan is associated with its chelating effect on metals and oligo-elements. Bacterial growth increases when metals and oligo-elements are present, and their absence results in bacterial death.

Kanatt et al. prepared composite films using chitosan and polyvinyl alcohol (PVA) incorporated with mint and pomegranate peel extracts. The results indicate that the tensile strength of the composite films improved by up to 41.07 ± 0.88 MPa when extracts were added without change in their puncture strength. They also reported that the extracts showed good antioxidant properties and antibacterial activity against *S. aureus* and *Bacillus cereus* [118].

**Figure 4:**
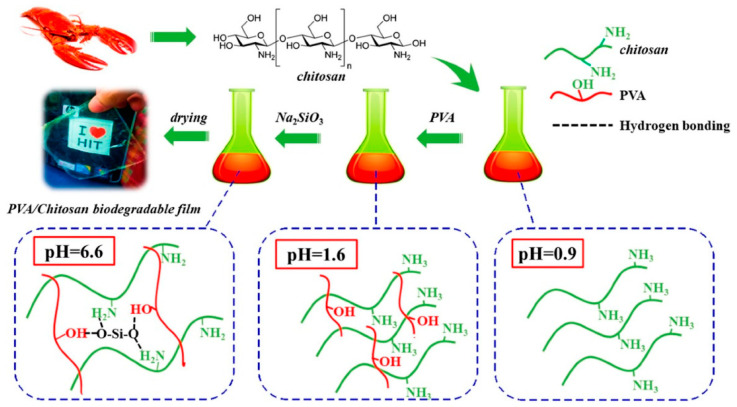
Polyvinyl alcohol/chitosan (PVA/CS) composite film in situ enhanced with silicon dioxide (SiO_2_) [119]. Reproduced with the permission of Elsevier.

Nevertheless, chitosan films have several disadvantages, such as poor mechanical properties and rapid dissolution in acidic solutions, which directly affect the quality of food-packaging films. Yu et al. synthesized a biodegradable composite film of chitosan, polyvinyl alcohol (PVA), and silica, as shown in Figure 4. The tensile strength of the chitosan/PVA composite films was improved by 45% (44.12 MPa) because of hydrogen bonds between silica and PVA or chitosan, and when a concentration of 0.6 wt % silicon dioxide (SiO_2_) was used. They also mentioned that when SiO_2_ was used, oxygen permeability and the moisture content of the composite film decreased. The biodegradation test confirmed that after 30 days in soil, the weight of the composite decreased to 60%. They also confirmed that the composite film is ideal for food packaging because it is high-performance, low-cost, and biodegradable [119].

### 6.5. Chitosan Nanoparticles

Chitosan nanoparticles are one of the excellent biopolymer-derived nanomaterials to be used in food packaging applications. The antibacterial activity of chitosan nanoparticles was ascribed to electrostatic interactions between the positively charged amino groups on chitosan and the negatively charged bacterial cell membranes. chitosan nanoparticles are better antimicrobial agents as compared to the chitosan itself [120]. Furthermore, quaternary chitosan nanoparticles are more effective against microorganisms because of the presence of quaternary ammonium ions [121].

**Figure 5:**
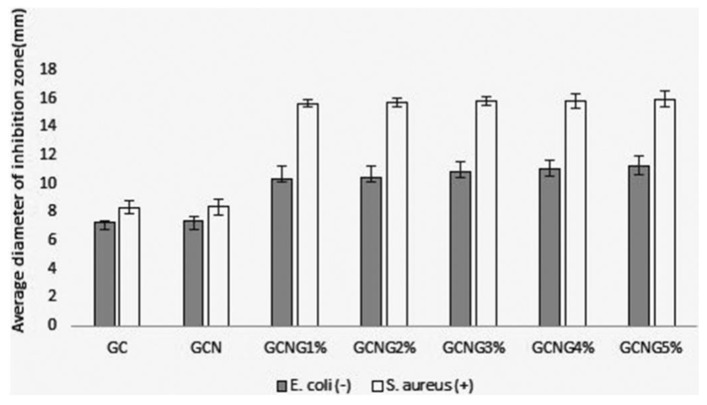
Antimicrobial properties of films derived from tragacanth gum (G)/nano chitosan (C) (GC), tragacanth gum (G)/nano chitosan (C)/alumina nanoparticles (N) (GCN), and GCN films with different (1–5%) concentrations of grape extract anthocyanin (G) (GCNG 1–5%) [122]. Reproduced with permission from Elsevier.

**Figure 6:**
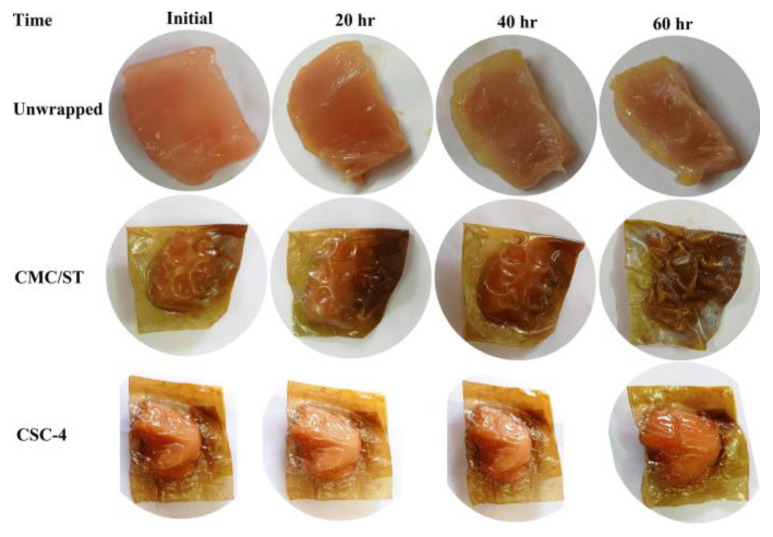
Response of unwrapped chicken with control film derived from carboxymethyl cellulose (CMC) and starch (ST) (CMC/ST), and carboxymethyl cellulose (CMC) and starch (ST) polymers reinforced with the incorporation of 9 wt% chitosan nanoparticles (CS NPs) (CSC-4) film on the freshness of chicken and spoiled chicken [124]. Reproduced with permission from Springer Nature.

In a recent study, chitosan nanoparticles were used to synthesize tragacanth gum/grape extract anthocyanin-derived films [122]. The films exhibited strong antibacterial activity against *E. coli* and *S. aureus*, as presented in Figure 5. This study indicated that the film has the potential to be used as a packaging material in the food industry. In another study, cinnamaldehyde-encapsulated chitosan nanoparticle-derived films were developed for meat preservation. The antimicrobial properties of the films were examined after 20 days, revealing a significant reduction in the total aerobic count, total coliform count, and growth potential of *Listeria monocytogenes* [123]. Amaregouda and Kamanna reported novel carboxymethylcellulose/starch-based nanocomposite films incorporating chitosan nanoparticles using a solution casting technique. These results indicate that the films had excellent antioxidant properties, with more than 90% cell viability. Additionally, the films showed the potential to prolong the shelf life of chicken meat at room temperature by up to 56 °h, as shown in Figure 6 [124].

**Figure 7:**
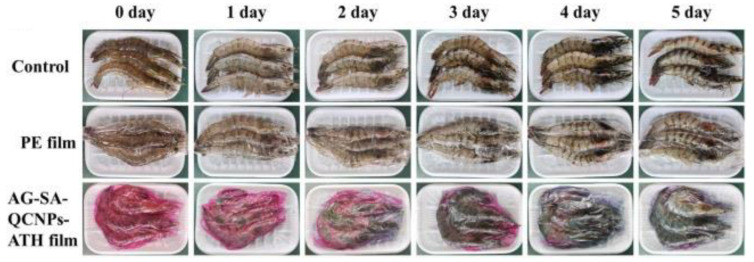
Pictures of shrimp preservation showing the control group (first row), polyethylene cling film (PE) packaging group (second row), and multifunctional films formed from edible agar (AG) and sodium alginate (SA), quercetin-loaded chitosan nanoparticles (QCNPs), and anthocyanin-rich purple sweet potato extract (ATH)AG-SA-QCNPs-ATH film group (third row) [126]. Reproduced with the permission of Elsevier.

Santhosh et al. used chitosan nanoparticles to synthesize jamun seed starch- and tamarind kernel xyloglucan-derived films. Chitosan nanoparticles with 3% *w*/*w* addition greatly enhanced the mechanical properties. The films showed excellent antimicrobial activity against *Bacillus cereus* and *Escherichia coli*. This study suggests that jamun seed starch/tamarind kernel xyloglucan films reinforced with chitosan nanoparticles are potential food packaging materials [125]. Using a combination of sweet potato extract and quercetin-loaded chitosan nanoparticles in an agar/sodium alginate polymer matrix, a film that served as an antibacterial material with a pH-sensitive indicator to monitor and preserve shrimp freshness was developed. The developed film had greater UV-blocking, good antioxidant and antibacterial properties, and excellent mechanical and water vapor barrier properties. The results indicated that shrimp packaged in this film maintained its freshness for up to 36 h during refrigerated storage, as shown in Figure 7. This multifunctional film can therefore be used in intelligent packaging that not only provides real-time monitoring but also effectively preserves the freshness of animal-based foods [126]. Fan et al. reported that films of gliadin-carboxymethyl chitosan composite nanoparticles (GCNPs) co-encapsulated natamycin (Nata) and theaflavins (TFs) for food packaging applications. The incorporation of Nata/TFs-GC NPs serves as a functional additive that enhances the optical and mechanical properties, water-blocking capabilities, and antifungal and antioxidant activities of the films. An in vivo test demonstrated the potential use of a functional film to inhibit the growth of *Aspergillus niger* on cheese [127]. Dos et al. prepared edible films from pectin incorporating chitosan nanoparticles, polysorbate 80 (T80), and garlic essential oil. The results demonstrate the effects of film hydrophilicity, which can alter the antimicrobial properties of the films. The films showed excellent antibacterial properties against *S. aureus*; only the films containing garlic essential oil showed inhibition through contact. For *E. coli*, the films with chitosan nanoparticles showed inhibition when they were in direct contact with the culture medium. These results suggest a potential alternative for creating stable antimicrobial nanoparticles for use in innovative food packaging [128].

## 7. Applications of Antimicrobial Packaging in Foods

Antimicrobial agents have long been used in food-related applications, especially food packaging [129] which includes increasing the shelf life of packed food and preventing the growth of microorganisms. This ensures food safety by preventing the proliferation of specific harmful organisms that come into direct contact with food packaging surfaces, such as cheese and meat, or liquids, such as meat exudates and milk. Antimicrobial packaging has significant potential to reduce the presence of harmful microorganisms in processed foods. The incorporation of antimicrobial agents into the polymer matrix is determined by various factors, such as the agent’s chemical composition, mode of action, spectrum of activity, rate of bacterial growth, and the physiological conditions of the targeted microorganisms. A crucial factor in this process is diffusion kinetics, which determines how the antimicrobial agents are released from the polymer. [5]. Moreover, antimicrobial packaging can possess sanitizing and self-sterilizing properties [15]. The activity of antimicrobials attached to polymers depends on the mode of action of the specific microorganism. Food components may lower antimicrobial activity by restricting diffusion from the polymers, nutrient-rich components, or media such as sulfates, lysine, and other sulfur-containing amino acids, such as in silver-substituted zeolites; therefore, the most practical application appears to be for nutrient-deficient beverages such as mineral water and tea. In plastic films, other polymers, such as triclosan, have high activity in growth media and low activity in foods [130].

In packaging materials, the concentration of the antimicrobial effect depends on factors such as the film thickness and polymer activity. Antimicrobial food packaging requires careful consideration of aspects such as thickness and physical and mechanical properties. In addition, the addition of plant extracts contributes to the color and opacity of the polymers [131], whereas the use of sorbates reduces the transparency of polyethylene films [132]. In chitosan-coated LDPE, the oxygen and water vapor transmission rates increased. The addition of specific antimicrobials to polymer complexes also changes the properties of polymers [133]. During polymer processing, various polymer additives, such as stabilizers, plasticizers, lubricants, and fillers, can negatively affect the antimicrobial activity of the polymer. These additives may change the polymer conformation, altering the diffusion or directly interacting with antimicrobials such as glycerol (plasticizer), which has a negative effect on lysozyme-incorporated cellulose triacetate [15].

## 8. Conclusions and Future Trends

The growing interest in antimicrobial packaging among researchers and industries is due to its capacity to enhance product safety and quality. Consumers in today’s fast-paced world are increasingly demanding fresh and minimally processed foods that are free from food-borne pathogens and microorganisms which can cause food deterioration and spoilage, leading to lower product quality and a shorter shelf life. Antimicrobial packaging systems can inhibit the growth of harmful microorganisms, thereby preserving the quality, freshness, and safety of food. Moreover, these systems prevent chemical contamination from conventional packaging materials, which can degrade the shelf life and barrier properties of food packaging materials.

Antimicrobial packaging is a significant application of active packaging, as it has a considerable influence on prolonging shelf life and ensuring food safety. Despite these benefits, creating antimicrobial packaging using eco-friendly materials with adequate mechanical and barrier properties while maintaining affordability continues to be a difficult task for both industries and consumers. The integration of nanostructures or nanoparticles in green packaging presents a promising solution for producing antimicrobial food packaging with enhanced mechanical properties. Based on this literature review, it is necessary to emphasize the importance of conducting future research in two essential areas. First, it underscores the crucial need to develop controlled release mechanisms for antibacterial agents using nanomaterials. This approach has great potential for achieving long-lasting antibacterial properties in food packaging. Second, the design and development of antimicrobial agents containing nanoparticles specifically tailored for food packaging materials. These agents should effectively eliminate microbes while ensuring minimal toxicity in packaged foods.

## Figures and Tables

**Table 1:** Examples of some promising antimicrobial agents for antimicrobial food packaging systems [18,41,42].

Classification	Antimicrobial Agents and Biopolymers
Organic acid/acid salts	Acetic, citric, sorbic, malic, lactic, and succinic acid, sodium benzoate, and potassium sorbate
Para benzoic acids	Ethanol
Bacteriocins/enzymes	Nisin, pediocin, subtilin, lacticin Lysozyme, lactoperoxidase, glucose oxidase
Fatty acids/esters	Laurie acid, palmitoleic acid, Gycerol mono laurate
Chelating agents/metals	EDTA and lactoferrin Copper, silver, and zirconium
Polysaccharide	Chitosan and Starch
Phenolics	Catechin, cresol, and hydroquinone
Plant/spice extracts/plant volatiles	Grape seed extract, grapefruit seed extract, rosemary oil, oregano oil, basil oil, and other herbs/spice oils Cinnamaldehyde, thymol, terpineol, Allyl isothiocyanate, eugenol, and pinene

**Table 2:** Antimicrobial agents and their applications in food packaging systems.

Antimicrobial Agents	Packaging Materials	Food	Microorganism	References
**Natural Extracts**				
(a) Grapefruit seed extract	LDPE, PLA/TPS PCL, Chitosan	Minced fish paste Salmon	*E-coli* and *Listeria monocytogenes* *E-coli*, *Psedomonas aeruginosa*	[51] [52]
(b) Clove extract	Chitosan-gelatine LDPE	Chilled pork Pork, culture media	*E.Coli*, *Psedomonas aeruginosa* *E. coli*, *S. Cervisiae*	[53] [54]
(c) Coptis chinesis extract	LDPE, paper	Ground beef	*E. coli*	[55]
(d) Horse radish extract	Chitosan, paper	Pork, fish fillet	*E. coli*	[56]
**Essential Oils**				
(a) Clove essentials oils	Chitosan Gelatin-chitosan, cypurs, cintronella	Dry cakes Cold fish fillets	*E. coli* and *S. aureus* *Aspergillus niger*, *Bacillus coagulans*	[57] [54]
(b) Oregano essential oil	Gelatin Starch Whey and milk protein	Grass carp fillets Fresh beef, chicken breast	*Pseudomonas*, *Aeromonas* *Zygosaccharomyces bailii* *Pseudomonas* spp.	[58] [59] [60]
(c) Cinnamon essential oil	Chitosan/PVA Chitosan film, active paper	Mangoes Rainbow trout, sliced bread	*E. coli* and *Staphylococcus aureus* and *C. lagenarium* *Oncorhynchus mykiss*, *Rhizopusstolonifer*	[61] [62]
**Organic Acids/Anhydride**				
(a) Benzoic acid	Agarose	Fish	*E. coli* and *Staphylococcus aureus*	[63]
(b) Sorbates	PBAT/TPS LDPE, MC/chitosan	Fresh noodles Cheese, chicken breast	*Aspergillus niger* and *Rhizopus* sp. Yeast, mold	[64] [65]
(c) Sorbic anhydride	PE	Culture media	Mold, *Saccharomyce S.cerevisiae*	[66]
**Enzymes**				
(a) Nisin, EDTA, lysozyme	SPI, Zein	Culture media	*E. coli*, *Lactobacillus plantarum*	[67]
(b) Immobilized lysozyme	PVOH, nylon, cellulose acetate	Culture media	Lysozyme activity test	[49]
**Bacteriocins**				
(a) Nisin	PE	Beef culture media	Total aerobes	[68]
(b) Nisin, citrate, EDTA	PVC, nylon, LLDPE	Chicken	*Staphylococcus aureus* *Sal. Typhimurium*	[69] [70]
(c) Nisin, lauric acid	Zein	Simulants	Migration test	[67]
**Biopolymers**				
(a) Chitosan.	Chitosan/peanut skin/pink peper	Chicken Strawberry	*psychrotrophic* *E. coli*	[71] [72]
(b) Chitosan, herb extract	Chitosan/paper LDPE	Culture media	*E. coli*, *S.cerevisiae*, *Fusarium oxysporum*	[73]
**Fungicides**				
(a) Imazalil	LDPE, PE	Bell paper, cheese	Molds	[74]
(b) Benomyl	Ionomer	Culture media	Molds	[75]
(c) Sulfur dioxide		Grape juice	Yeast	[76]
**Others**				
(a) Ageless^®^	Sachet	Bread	Molds	[77]
(b) Ethanol	Silica oxide sachet	Bakery	Molds	[78]
**Metal/Metal Oxide Nanoparticles**				
(a) Silver	Chitosan/PVA	Strawberry	*E. coli*	[79]
(b) Copper/copper oxide	TPS	Meat fresh products	*E. coli* and *S. aureus*	
(c) Zinc oxide	Starch/Chitosan Bacterial cellulose LDPE PVA-gelatin	Food packaging Culture media Culture media	*Staphylococcus aureus* *Bacillus subtilis* and *Enterobacter aerogenes* *Bacillus subtilis,* *E. coli*	[80] [81] [82]
(d) Titanium dioxide	PVA/CNC/Apple peel extract Chitosan	Cherry tomatoes Culture media	Fungi, Mold and bacteria *E. coli*, *Staphylococcus aureus*, *Candida albicans*	[83] [84]

LDPE—low-density polyethylene; HDPE—high-density polyethylene PE—polyethylene; PVC—polyvinyl chloride; PVOH/PVA—polyvinyl alcohol; MC—methyl cellulose; PLA—poly(lactide); TPS—thermoplasticized starch; CNC—cellulose nanocrystal.

**Table 3:** Commercial antimicrobial packaging for food applications [15,95,96].

Antimicrobial Compound	Tradename	Company Name	Packaging Forms
Silver substituted zeolite	AgIon^TM^ Novaron^®^	AgIon Technologies Inc. (Wakefield, MA, USA) Toagosei, Co. Ltd. (Tokyo, Japan)	Bulk food storage containers, paperboard cartons, plastic or paper food wraps, and milk containers
Triclosan	Microban^®^	Microban Product (Toronto, ON, Canada)	Deli wrap, reheatable food containers
Allyl isothiocyanate	WasaOuro	Lintec Corporation (Tokyo, Japan) Dry Company Ltd. (Tokyo, Japan)	Pressure-sensitive labels, sheets Sachets
Chlorine dioxide	Microsphere^TM^	Bernard Technologies Inc. (Aalen, Germany)	Produce storage bags, paperboard coating, rigid containers, pressure-sensitive labels
Carbon dioxide	Freshpax^TM^ Verifrais	Multisorb Technologies (Buffalo, NY, USA) SARL Codimer (Paris, France)	Sachets Sachets
Ethanol vapor	Ethicap^®^ Negamold^®^ Fretek^®^ Oitech^TM^	Freund (Tokyo, Japan) Nippon Kayaku (Chiyoda City, Tokyo)	Sachets Sachets Sachets
Glucose oxidase	Bioka	Bioka Ltd. (Kantvik, Finland)	Sachets

**Table 4:** Antimicrobials covalently and ionically immobilized in a polymer matrix, (modified from [15,99]).

Functional Polymer Support	Antimicrobials
Ionomeric films	Benomyl Benzoyl chloride Becteriocin
Polystyrene	Lysozyme Synthetic antimicrobial peptides
Polyvinyl alcohol	Lysozyme
Nylon 6,6 resins	Lysozyme

## Data Availability

Not applicable.
